# Bi‐exponential modeling derives novel parameters for the critical speed concept

**DOI:** 10.14814/phy2.13993

**Published:** 2019-02-19

**Authors:** Mark Kramer, Rosa Du Randt, Mark Watson, Robert W. Pettitt

**Affiliations:** ^1^ Human Movement Science Department Nelson Mandela University Port Elizabeth South Africa; ^2^ Psychology Department Nelson Mandela University Port Elizabeth South Africa; ^3^ Rocky Mountain University of Health Professions Provo Utah

**Keywords:** $\dot{V}O_{2}\max$, 3‐min all‐out exercise test, critical power, shuttle running, speed reserve

## Abstract

All‐out exercise testing (AOT) has emerged as a method for quantifying critical speed (CS) and the curvature constant (D′). The AOT method was recently validated for shuttle running yet how that method compares with linear running is unknown. In the present study, we utilized a novel bi‐exponential model that derives CS and D′ with additional new parameters from the AOT method. Fourteen male athletes (age = 21.6 ± 2.2 years; height = 177 ± 70 cm; weight = 83.0 ± 11.8 kg) completed a graded exercise test (GXT) to derive maximum oxygen uptake ($\dot{V}O_{2}\max$) and the average speed between gas exchange threshold and $\dot{V}O_{2}\max$ (sΔ50%), a linear AOT, and two shuttle AOTs. Measurement agreement was determined using intraclass correlation coefficient (ICC α), typical error (TE), and coefficient of variation (CV). The *y*‐asymptote ($S_{0}$) of the speed‐time curve (3.52 ± 0.66 m·sec^−1^) did not differ from sΔ50% (3.49 ± 0.41 m·sec^−1^) or CS (3.77 ± 0.56 m·sec^−1^) (*P* = 0.34). Strong agreement was observed for estimates of CS (ICC α = 0.92, TE = 0.18 m·sec^−1^, and CV = 5.7%) and D′ (ICC α = 0.94, TE = 16.0 m, CV = 7.6%) with significant (*P* < 0.01) correlations observed between $\dot{V}O_{2}\max$ and CS and between $S_{0}$ and $\dot{V}O_{2}\max$ (*r* values of 0.74 and 0.84, respectively). The time constant of the decay in speed ($\tau_{d}$) and the amplitude between maximal speed and $S_{0}$ ($A_{d}$) emerged as unique metrics. The $A_{d}$ and $\tau_{d}$ metrics may glean new insights for prescribing and interpreting high‐intensity exercise using the AOT method.

## Introduction

High‐intensity running is characterized by a hyperbolic relationship between running speeds and performance times (*t*_LIM_) (Hill 1925). The *t*_LIM_ (*x*‐axis) associated with running different distances (*y*‐axis) may be used to derive critical speed (CS, slope) and the finite capacity for running speeds exceeding CS (D′, intercept) (i.e., the distance‐*t*_LIM_ model, or D‐*t*_LIM_) (Fukuba and Whipp 1999). The CS parameter identifies a robust fatigue threshold separating sustainable from nonsustainable running speeds, or the “heavy” and “severe” domains of high‐intensity exercise (Poole et al. 2016). Alternatively, using speed (*y*‐axis) and the inverse time (*x*‐axis), or the speed‐inverse time (S‐1/*t*_LIM_) model, the CS and D′ are the intercept and slope, respectively (Fukuba and Whipp 1999). When algebraically transformed, a given running speed (m·sec^−1^) in the severe‐intensity domain can be resolved using:

(1) $$\text{Speed}=(D^{\prime }/\text{time})+\text{CS}$$

The D‐*t*_LIM_ and S‐1/*t*_LIM_ methods for determining CS and D′ required time trials of three or more distances. Alternatively, the CS and D′ parameters can be derived more expediently using the 3‐min all‐out exercise test (AOT) (Pettitt et al. 2012; Broxterman et al. 2013). With the AOT method, the CS is derived theoretically by expending D′ completely via all‐out running within a span of 150 sec (i.e., D′ = zero in eq. (1)), resulting in an average speed during the last 30 of 180 sec equaling and predicting CS. With these results, the speed for a given *t*_LIM_ can be estimated from single AOT in the severe domain. Likewise, the *t*_LIM_ associated with a given distance (D) can be derived from the AOT using:

(2) $$t_{\mathrm{LIM}}=\left(D-D^{\prime }\right)/\text{CS}$$

With all‐out sprints of very short durations and distances (e.g., 40 m dash), the speed‐time curve is mono‐exponential; however, all‐out running of longer durations yields a time‐dependent decay in speed resulting in the appearance of a second exponent of the speed‐time curve (Morin et al. 2006; Heck and Ellermeijer 2009). The speed–time relationship of an AOT resembles as bi‐exponential relationship; yet, to our knowledge, no one has attempted to evaluate the AOT method in such a manner.

The AOT method has been validated against the D‐*t*_LIM_ and S‐1/*t*_LIM_ methods for all‐out shuttle running and the subsequent applications of equations 1 and 2 for shuttle running (Saari et al. 2017). In that study, repetitive bursts of accelerations and decelerations occurred with the 180° turns; however, the overall speed‐time curve for the AOT appeared bi‐exponential. Moreover, comparisons between continuous and shuttle running were not conducted. With bi‐exponential modeling, the descending curve (second component) should have an asymptote that is equivalent to CS and in close proximity to 50% of the difference (s50%Δ) of the speed evoking gas exchange threshold and maximum oxygen uptake ($\dot{V}O_{2}\max$) during a graded exercise test (GXT) (Pettitt et al. 2012). The integral of the two components of the speed‐time curve, above CS, hypothetically compose the D′; yet, additional performance parameters of interest from exponential modeling may be derived. Therefore, the purpose of the study was to evaluate a novel bi‐exponential model for quantifying performance elements of the AOT for both linear and continuous, all‐out shuttle running.

## Materials and Methods

### Experimental overview

Subjects visited the testing facility on five separate occasions over a 3‐week period with each visit separated by at least 48 h. Visit 1 entailed familiarizing subjects with the testing procedures prior to the start of experimentation. Visit 2 was used to conduct a GXT with verification bout with the key metrics being Δ50% and $\dot{V}O_{2}\max$. Visits 3 through 5 were allocated for conducting the three separate AOTs in counterbalanced succession to avoid an order‐effect. One AOT was the standard 3‐min all‐out running test (Pettitt et al. 2012; Broxterman et al. 2013) whereas the other AOTs involved continuous, shuttle running of 25 and 50 m distances. Each AOT was completed on the same outdoor 400‐m track during a season and time of day with ambient temperatures and low winds. All AOT data were evaluated using the original methods along with our novel bi‐exponential model.

### Subjects

A total of 14 male field athletes (soccer, *n* = 3; rugby, *n* = 11) volunteered and completed all phases of the study. The Nelson Mandela University (NMU) research ethics committee for human test subjects, in accordance with the Code of Ethics of the World Medical Association (Declaration of Helsinki), approved all procedures. All subjects provided written consent after having the experimental procedures explained both verbally and in written format. Subjects were recruited from the NMU first team soccer and rugby clubs, were apparently healthy, had a minimum of 1‐year competitive playing experience at the relevant level, were not taking any medications, and were uninjured at the time of testing. The subjects had the following characteristics (mean ± SD): age = 21.6 ± 2.2 years, height = 177 ± 70 cm, and weight = 83.0 ± 11.8 kg.

### Laboratory‐based GXT with exhaustive verification bout

Prior to the GXT, subjects completed a 5‐min warm‐up at 6–8 km·h^−1^, followed by a 5‐min rest period during which subjects completed dynamic stretches. The GXT began at 8 km·h^−1^ at an incline of 1% grade on a motorized treadmill (Woodway 4Front, USA) to replicate the equivalent $\dot{V}O_{2}$ cost for outdoor running (Jones and Doust 1996). Treadmill speed was increased by 1 km·h^−1^ every min until exhaustion as defined by the subject straddling the treadmill belt. Inspired and expired gas volume and concentrations were sampled breath‐by‐breath using an automated open circuit spirometry (Metamax 3B, Cortex Biophysik). The system was calibrated prior to each test per the manufacturer's instructions. Gas exchange data were reduced to 15‐s averages for the estimation of gas exchange threshold (GET) using the V‐slope method (Beaver et al. 1986). The speeds evoking GET and the highest $\dot{V}O_{2}$ value in the GXT were interpolated at 1‐min preceding the sample and used to calculate sΔ50% (Pettitt et al. 2012). A 3‐min recovery following the GXT preceded the exhaustive verification bout carried out at an intensity equivalent to two stages preceding end stage (Pettitt et al. 2012). “True” $\dot{V}O_{2}\max$ was the highest value obtained with < 3% difference between the highest $\dot{V}O_{2}$ values observed for the GXT and verification bout (Pettitt et al. 2013).

### All‐out bout procedures

A standardized warm‐up of a 400‐m lap of jogging, dynamic stretches and build‐up sprints, and a 5‐min rest period preceded each AOT. The linear AOT was recorded using a wrist‐worn global positioning system (GPS) device sampling at 1 Hz (Forerunner Model 305, Garmin, Taiwan). The shuttle AOTs were video recorded without panning from an elevated position at 100 Hz (Cyber‐shot DSC‐RX10 MK III, Sony, USA). Video files were exported to a motion analysis software package (Tracker 4.11.0, Open Source Physics) calibrated to known distances along with setting the origin of the reference frame to the starting cone for the shuttle run. The automated motion tracking feature was utilized to increase the accuracy of the digitization process by tracking the motion of the subject's head throughout the entirety of the all‐out run. Although markers were placed at the approximated center of mass (COM) of the body during the pilot testing, that marker ineffectively tracked the COM due to movement of the arms which, when coupled with the frequent turns, increased the potential for digitization errors. An apparent deformation of the body during turning maneuvers was observed whereby the head aligned more closely with the COM. The digitization process provided near instantaneous displacement information for each athlete. Displacement data were differentiated to obtain speed and filtered using a forth order zero‐lag Butterworth filter with a cutoff frequency of 2–6 Hz (Winter 2009).

### Analysis of AOT bouts

**Figure 1:**
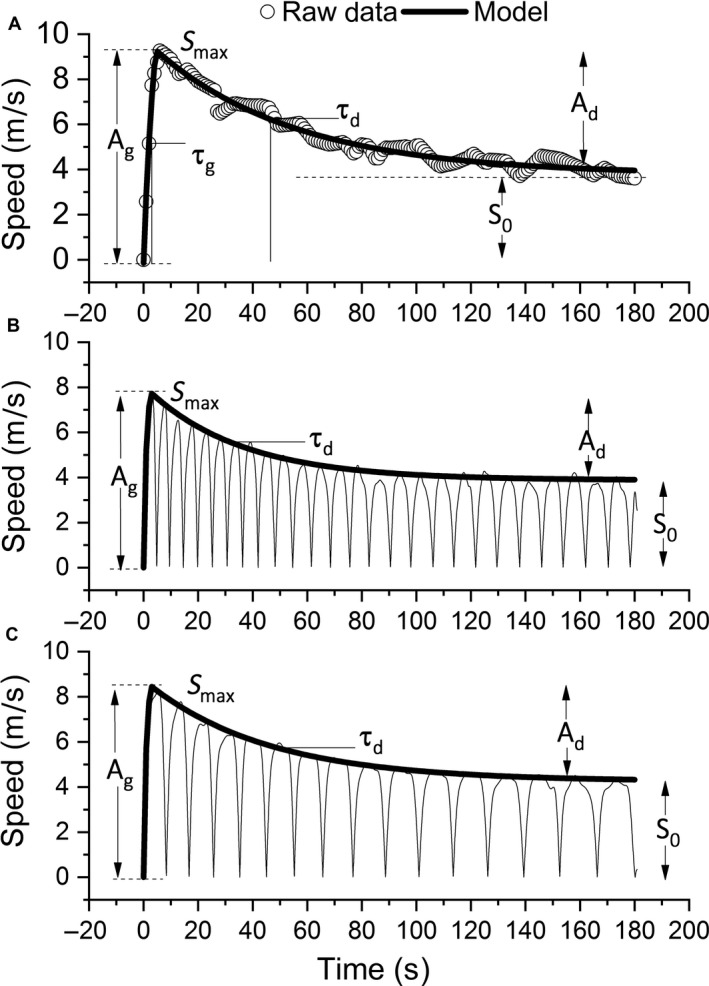
Linear (Panel A), 50 m (Panel B), and 25 m (Panel C) shuttle all‐out exercise tests (AOT). Parameters of the $S^{\prime }$model are shown with A_*g*_ representing the amplitude of all‐out speed to $S_{\max}$ (i.e., peak speed in the AOT), $t_{c}$ represents the time to reach $S_{\max}$, $S_{0}$ represents the y‐asymptote or surrogate measure of critical speed, $A_{d}$ represents the amplitude of decay between $S_{\max}$ and $S_{0}$, and $\tau_{d}$ represents time to reach a ~63% decrease in the speed between $S_{\max}$ and $S_{0}$.

The CS (m·sec^−1^) for the AOTs was calculated using the average speed of the last 30 of the 180 sec. The D′ (m) was calculated by subtracting CS from the average speed of the initial 150 sec (m·sec^−1^), multiplied by 150 sec (Pettitt et al. 2012). Both the GPS and video extracted speed data were exported and subjected to a novel bi‐exponential model (OriginPro, 2017 [version 94E], OriginLab, USA). Data were interpolated to give one value per second and time aligned to the start of the test. The speed‐time curve was then fitted using the following equation:

(3) $$S\left(t\right)=\left\{\begin{array}{cc} S_{0}+A_{d}+A_{g}\cdot \left(e^{-t_{c}/\tau_{g}}-e^{-t/\tau_{g}}\right) & t\leq t_{c}\\ S_{0}+A_{d}\cdot e^{-\left(t-\;t_{c}\right)/\tau_{d}} & t>t_{c} \end{array}\right.$$

where t is the time, S(t) is the speed at a given time, $S_{0}$ is the y‐asymptote or the surrogate metric of CS, $A_{g}$ is the growth amplitude of the exponential, $A_{d}$ is the decay amplitude of the exponential, $t_{c}$ is the time offset between exponential growth and decay, $\tau_{g}$ is the time constant of the exponential growth term and $\tau_{d}$ is the time constant of the exponential decay term. In practical terms, maximum speed ($S_{\max}$), or the apex of D′, is derived by summing the $S_{0}$ and $A_{d}$ terms, the $t_{c}$ reflects the time to $S_{\max}$, $\tau_{d}$ reflects the rate of decline in speed toward $S_{0}$. Figure 1, panel A, provides a graphical representation of these parameters.

The “gain” between $S_{0}$ to $S_{\max}$ provides a speed reserve for running speeds exceeding $S_{0}$. To calculate that speed reserve, as a percentage of total speed, a fatigue index (FI) was derived using:

(4) $$\text{FI}\;\left(\%\right)=100\cdot \left(\left(S_{\max}-S_{0}\right)/S_{\max}\right)=100\cdot \left(A_{d}/S_{\max}\right)$$

Stated specifically, smaller FI percentage values would identify athletes with lower relative indices of fatigability, or athletes with $S_{0}$ values in closer proximity to $S_{\max}$ and a relative propensity for engaging in endurance activities.

### Statistical analyses

Summary statistics are reported as mean ± SD. All data were assessed and conformed to normality as identified by the Shapiro–Wilk test. Multiple linear regression was used to derive a composite metric of $S^{\prime }$ (see Eqn 5 in Results) whereby that value versus D′ was compared using a paired samples *t* test. Separate analyses of variance with repeated measures were used to evaluate differences of the CS and D′ metrics between the three AOTs, whereas as Δ50% was added as a 4th level of the independent variable for CS. The Scheffé test was used when post hoc exploration was necessary. In cases where measurement agreement between surrogate and actual measures was of interest, we report the intraclass correlation coefficient (ICC α), typical error (TE), and coefficient of variation (CV%) (Hopkins 2000). Pearson‐product moment correlation coefficients (*r*) were used to quantify the relationships or lack thereof between metrics with different units of measurement. The level for rejecting null hypotheses was set at *P* < 0.05.

## Results

**Figure 2:**
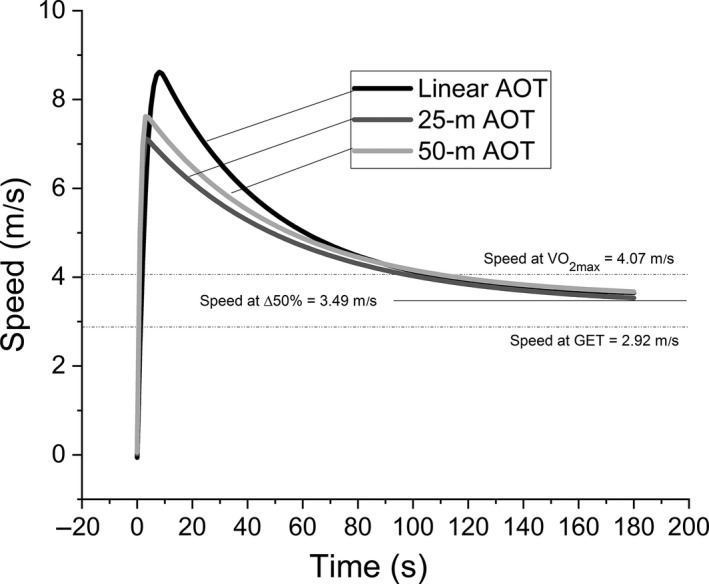
Comparison of the $S^{\prime }$model between the linear, 50 m, and 25 m shuttle all‐out exercise tests. Take note of the between‐condition decline in $S_{\max}$ and proximity of $S_{0}$ relative to the Δ50% parameter derived from the graded exercise test.

**Figure 3:**
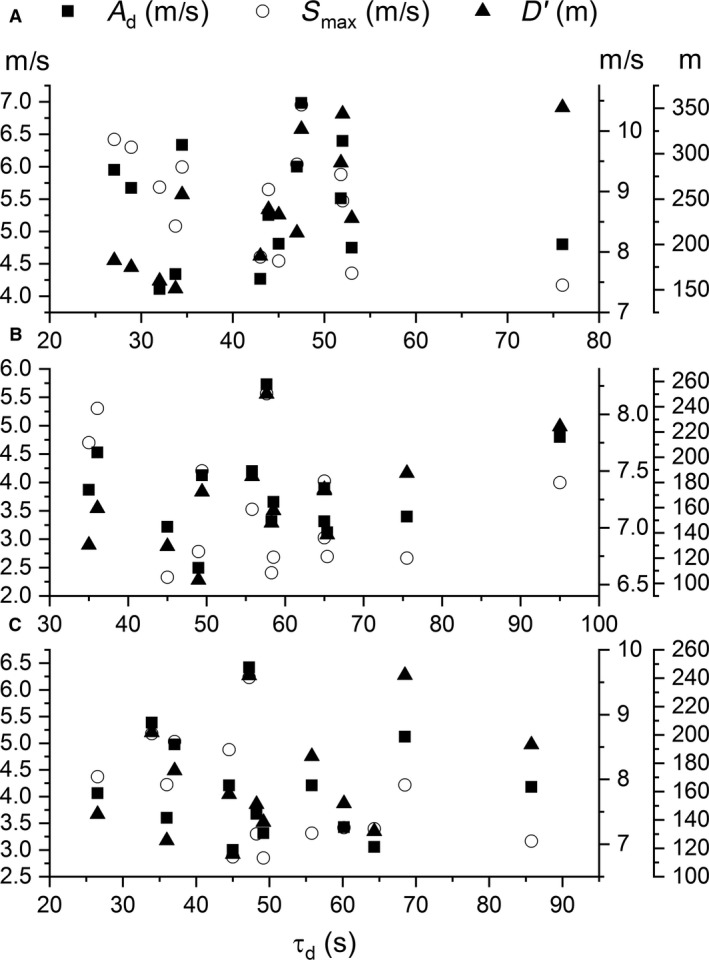
Scatterplots comparing $\tau_{d}$ (*x*‐axes) versus $A_{d}$ (left *y*‐axis) and $S_{\max}$ (right *y*‐axis). Panels A, B, and C represent the linear, 50 m, and 25 m shuttle running all‐out exercise tests, respectively.

The fit of the bi‐exponential model was very strong (*r* value M ± SD) for the linear (0.94 ± 0.03), 25 m shuttle (0.98 ± 0.02), and 50 m (0.97 ± 0.02) shuttle AOTs (Fig. 1). A higher $S_{\max}$ value was achieved for the linear versus shuttle AOTs (Fig. 2), and equally, times to $S_{\max}$ (i.e., $t_{c}$) were faster for the shuttle AOTs versus the linear AOT (Table 1). Similarly, as shown in Table 2, greater depreciation of high‐intensity running, as measured by the FI, was observed for the linear versus the shuttle AOTs; yet, interestingly, no significant differences were observed for either the $\tau_{d}$ or $S_{0}$ parameters. Also noteworthy were the nonsignificant, linear correlations (range of *r* values = 0.11–0.53, *P* > 0.05) between $\tau_{d}$ versus $A_{d}$ (closed squares) and $\tau_{d}$ versus $S_{\max}$ (open triangles) (closed triangles) (Fig. 3).

**Table 1:** Parameter estimates from the S′‐model

Parameter	Linear AOT	50 m AOT	25 m AOT	ANOVA Statistics (F, *P*)
$S_{\max}$ **(m·sec** ^**−1**^ **)**	8.88 ± 0.91^b^, *** ^,^ c, **	7.76 ± 0.84^a^, **	7.18 ± 0.54^a^, ***	F [17.39], *P* < 0.001
$t_{c}$ **(sec)**	6.42 ± 1.96^b^, *** ^,^ c, ***	3.10 ± 0.82^a^, ***	2.84 ± 0.37^a^, ***	F [35.83], *P* < 0.001
$\tau_{d}$ **(sec)**	43.96 ± 12.73	50.16 ± 15.69	57.90 ± 15.61	F [3.14], *P* = 0.054
$A_{d}$ **(m·sec** ^**−1**^ **)**	5.37 ± 0.89^b^, *** ^,^ c, **	4.19 ± 0.98^a^, **	3.83 ± 0.82^a^, ***	F [11.19], *P* < 0.001
FI **(%)**	60.34 ± 6.92	53.50 ± 7.99	53.00 ± 8.11	F [3.98], *P* = 0.027
$S_{0}$ **(m·sec** ^**−1**^ **)**	3.52 ± 0.66	3.57 ± 0.51	3.35 ± 0.48	F [0.61], *P* = 0.548
$S^{\prime }$ **(m)**	237.20 ± 61.27^b^, ** ^,^ c, **	168.21 ± 39.02^a^, **	166.28 ± 37.99^a^, **	F [10.52], *P* < 0.001

Values are mean ± SD. $S_{\max}$, S_0_ + *A*_d_; maximum speed; $t_{c}$, time delay to $S_{\max}$; $\tau_{d}$, decay time constant; $A_{d}$, decay amplitude; FI, fatigue index; $S_{0}$, critical speed; $S^{\mathrm{prime}}$, speed reserve. ^a^significantly different from linear AOT, ^b^significantly different from 25‐m AOT, ^c^significantly different from 50‐m AOT,

*P < 0.05, **P < 0.01, ***P < 0.001.

**Table 2:** Multiple linear regression for S′

Parameter	Linear AOT	50 m AOT	25 m AOT
*r*	0.91	0.97	0.95
*r* ^2^	0.83	0.94	0.90
Adjusted *r* ^2^	0.80	0.93	0.88
SEE (m)	30.31	10.43	13.25
SEE (%)	12.73	6.20	7.95
F (statistic, *P*)	26.60 (*P* < 0.001)	90.91 (*P* < 0.001)	49.41 (*P* < 0.001)
FI ($\beta_{1}$, *P*)	5.21 (*P* = 0.003)	4.71 (*P* < 0.001)	3.82 (*P* < 0.001)
$\tau_{d}$ ($\beta_{2}$, *P*)	2.83 (*P* = 0.003)	0.38 (*P* = 0.068)	0.80 (*P* = 0.008)
Intercept ($\beta_{3}$, *P*)	−200.84 (*P* = 0.020)	−102.98 (*P* < 0.001)	−82.00 (*P* = 0.008)

*r*, correlation coefficient; *r* ^2^, coefficient of determination; SEE, standard error of the estimate; FI, fatigue index; $\tau_{d}$, decay time constant.

Using the FI and $\tau_{d}$ metrics from equation 4, multiple linear regression was used to derive the following equation:

(5) $$S^{\prime }=\;\beta_{1}\text{FI}+\;\beta_{2}\tau_{d}+\;\beta_{3}$$

where $S^{\prime }$ comprised the area under each component of the bi‐exponential curve above $S_{0}$ and the β ‐coefficients are presented in Table 2. Strong measurement agreement was observed between D′ (225.1 ± 59.8 m) and $S^{\prime }$ (238.2 ± 67.4 m) (ICC α = 0.94, TE = 16.0 m, CV = 7.6%) with no significant differences between measures (*t* = 2.17, *P* < 0.05).

No differences were observed between CS and $S_{0}$ between any of the AOTs; however, D′ and $S^{\prime }$ from the linear AOT exceeded metrics of the shuttle AOTs (Table 1). The speeds (m·sec^−1^) evoking GET (2.92 ± 0.36) and $\dot{V}O_{2}\max$ (4.07 ± 0.49) from the GXT were used to derive sΔ50% (3.49 ± 0.41). The sΔ50% did not differ from the linear CS (3.77 ± 0.56) (*P* = 0.42) or $S_{0}$ (3.52 ± 0.66) metrics (*P* = 0.99). The CS metric of the 25 m AOT was lower than the linear AOT (*P* = 0.01).

The relative $\dot{V}O_{2}\max$ values (mL·kg^−1^·min^−1^) between the GXT (44.1 ± 4.3) and the verification bout (43.9 ± 3.8) did not differ significantly (*t* = 0.70, *P* = 0.50) and exhibited strong measurement agreement (ICC α = 0.96, TE = 0.84 mL·kg^−1^·min^−1^, CV% = 1.90) for assessing “true” $\dot{V}O_{2}\max$. Strong correlations were observed between $\dot{V}O_{2}\max$ and CS (*r *=* *0.74, *P* < 0.01) along with $S_{0}$ (*r *=* *0.84, *P* < 0.01).

## Discussion

The bi‐exponential model introduced in this study provides a mathematical description of discrete elements of the D′, a measure of the finite capacity for high‐intensity running at speeds exceeding CS (Fukuba and Whipp 1999). As with the CS, the $S_{0}$ parameter was similar to the Δ50% observed in a GXT and correlated positively with “true” $\dot{V}O_{2}\max$. The bi‐exponential model provided a strong fit for both the linear and shuttle AOTs, whereby, both the raw and modeled data indicated lower measures of D′ for shuttle running using 3‐min all‐out bouts. Finally, our data indicate that the additional parameters of the FI and the $\tau_{d}$ from the bi‐exponential model, may offer new insight into physiological parameters alluded by the D‐*t*_LIM_ or S‐1/*t*_LIM_ models. Moreover, the coefficients located in Table 2, may be utilized with equation 5 to provide an accurate estimate of $S^{\prime }$ (i.e., our surrogate measure for D′) using the FI and $\tau_{d}$ metrics from an AOT and equation 4.

In the bi‐exponential model, $S_{\max}$ and $t_{c}$ are readily identified and provide comparisons of interest between linear and shuttle running. Specifically, lower $S_{\max}$ and faster $t_{c}$ parameters were observed for shuttle versus linear running, due to the necessity to accelerate and decelerate preceding each 180° turn. Although $S_{\max}$ and $t_{c}$ measured using the AOT procedure may change in response to an intervention, one should consider that different values would be derived from all‐out sprinting of shorter distances (e.g., peak sprinting in a 40‐m dash). That said, given recent evidence associating neuromuscular strength/cross sectional volume with W′ in cycling (Kordi et al. 2018), changes in the $A_{d}$ metric (Fig. 1) may be used to potentially detect neuromuscular strength adaptations and their contribution to D′ in running.

The 25‐m AOT presented with lower CS and D′ values in comparison to the linear AOT. The shorter distance and larger frequency or total count of 180° turns compared to the 50‐m AOT, limited that magnitude of the $S_{\max}$ parameter, and the average speed achieved within each shuttle. The interpretation of the lower CS in the 25‐m AOT is not necessarily attributed to a decline in the maximal aerobic steady‐state, but rather, a decline imposed simply by the need to decelerate on a repetitive basis (i.e., not sufficient time or distance to achieve a similar CS to that of the linear AOT).

The model of fit (e.g., *r* ^2^ value) for the $S^{\prime }$ equation (eq. 5) was slightly lower for the linear versus the shuttle AOTs (Table 2). A notable explanation for the trend is the stronger contribution of FI and $\tau_{d}$ to the $S^{\prime }$ metric in linear running. The $S_{\max}$ and FI metrics were larger, and the $\tau_{d}$ was shorter, for the linear AOT versus the shuttle AOTs suggesting that the higher speeds reached in linear running evoked more rapid decrements of metabolic energy. Despite a lower fit for the $S^{\prime }$ in the linear AOT, the estimate of D′ was not significantly different from $S^{\prime }$ with a low TE and CV%.

The FI metric was necessary for determining $S^{\prime }$ (Eqn 5), both of which denote the range of speeds above CS (i.e., the magnitude of the speed reserve; see Fig. 1). The FI metric differed for the linear versus the shuttle AOTs, due largely to the differences in $S_{\max}$. The FI metric, as introduced, provides a relative method of comparing the endurance capacity of two athletes (i.e., a smaller FI would be associated with a higher endurance athlete). What the FI also shows is that athletes with higher $S_{\max}$ values tend to experience greater speed decrements toward CS, a finding commensurate with a previous shuttle running study (Buchheit et al. 2010), but is uniquely captured within our model. Furthermore, a ratio of $S^{\prime }$ to total distance would quantify the total proportion of distance traveled supported predominantly by anaerobic energy sources (Pettitt 2012) based on the notion that $S^{\prime }$ is linked to the finite distance attainable within the severe‐intensity domain. When contextualized to the present study, relative anaerobic contributions of ~27%, ~21%, and ~20% were observed for the linear, 25‐m, and 50‐m AOTs, respectively; a finding on par with studies focusing on 800‐to‐1500 m running (Duffield et al. 2005a,b).

With the D‐*t*_LIM_ and S‐1/*t*_LIM_ models, the D′ represents a capacity. If D′ is wholly expended during high‐intensity running (i.e., speeds in the severe‐intensity domain), the speed associated with *t*_LIM_ of performances would decrease toward CS exponentially in accordance with the kinetic energy equation (Pettitt 2012). Such a proportion for partial expenditure of D′ may vary (e.g., engaging in high‐intensity interval training). Using the $S^{\prime }$ equation, a partial rate of expenditure for D′ can be quantified using the $\tau_{d}$ parameter.

The $\tau_{d}$ parameter delineates the rate at which ~63% of the speed at $S_{\max}$ declines toward $S_{0}$, where $S_{0}$ represents the surrogate of CS within the bi‐exponential model. When visualizing the AOT, such a comparison would be analogous to a decline of the apex of D′ toward CS (i.e., expending ~63% of the height of $A_{d},$ see Fig. 1). Thus, an athlete with large $S_{\max}$ and FI metrics might be quite attractive for the sport of rugby but less suitable for “sprinting” to the finish line at the end of a 5‐K foot race. Such a characteristic about an athlete's D′ cannot be detected using the D‐*t*_LIM_ and S−1/*t*_Lim_ models. Moreover, certain energetics models rely on the assumption that the utilization and recovery of D′ conform to a curvature constant (Ferguson et al. 2010; Skiba et al. 2014). On the contrary, our findings indicate that there was a fairly large standard deviation for $\tau_{d}$ suggesting there may be individual differences in the rates at which different people can utilize D′ (i.e., the utilization and recovery of D′ is not subject to a constant). Distinguishing $\tau_{d}$ as a distinctive parameter of high‐intensity performance is visualized by the lack of a correlation between $\tau_{d}$ versus $S_{\max}$ and $A_{d}$ (Fig. 3). Stated plainly, subjects with lower values for $\tau_{d}$ expend a greater proportion of D′ at the onset of exercise in comparison to subjects with higher values of $\tau_{d}$. Such differences would be more integral, and $\tau_{d}$ a more distinguishing feature, for team‐sport athletes versus endurance athletes with inherently lower D′ values. The parameters of $S_{\max}$, FI, and $\tau_{d}$ may therefore account for intervention‐specific differences linked to D′ that the D‐*t*_LIM_ and S‐1/*t*_LIM_ fail to capture. Moreover, because the linear AOT is already used regularly for the evaluation of aerobic fitness (Kramer et al. 2018) and the derivation of CS and D′ parameters, it is pertinent to note that the bi‐exponential model presented provides additional information that can be captured and tracked longitudinally; thereby, complementing an already robust assessment method.

The $\dot{V}O_{2}$ kinetics time constant (τ) is shown routinely as a growth constant for the total amplitude of an exponential curve of $\dot{V}O_{2}$ kinetics toward a “steady‐state” (Poole and Jones 2012). Similarly, in the present study, $\tau_{d}$ represents a decay constant toward the *y*‐asymptote (or $S_{0}$), a surrogate measure of CS, or a maximal “steady‐state” for running speed. The issue of pacing and inflating the estimate of CS with the AOT has been raised previously (Pettitt 2016; Saari et al. 2017). Using the bi‐exponential model, pacing could be potentially detected by evaluating $\tau_{d}$ representing a line of inquiry for future investigators.

Of considerable interest in our results are the physiological causes for the variability of $\tau_{d}$. Based on the variability of $\tau_{d}$ observed in our sample, we can deduce that ~63% of D′ was expended between ~30 and 60 s of all‐out exercise; the magnitude of which is quantified by $S_{\max}$ and FI. Such declines in high‐intensity running within this time frame are attributed to [PCr] (Jones et al. 2008) and muscle glycogen depletion (Miura et al. 2000); however, the rates of utilizing these substrates may be predicated on the availability of key enzymes such as creatine kinase and the lactate dehydrogenase isozyme favoring the production of lactate. Additionally, faster $\tau_{d}$ values may be partially attributed to the time‐dependent decline in pH and a less sufficient rate to remove hydrogen ions from the sarcoplasm (Jones et al. 2008). The physiological underpinnings ascribed to the modeled parameters presented here provide impetus for future research and the associated links to all‐out testing. The fact that the $\tau_{d}$ did not differ between the linear and shuttle AOTs would suggest the parameter is metabolic and less influenced by differences in biomechanical constraints imposed by shuttle versus linear running.

With running speeds exceeding the average speed of the initial 150 sec of all‐out sprinting, *t*_LIM_ associated with a partial expenditure of D′ could be estimated assuming a linear proportion D′ expenditure (i.e., 80% of D′ would produce a *t*_LIM_ * 0.8) (Pettitt et al. 2012). Such an assumption has led to the successful implementation of the CS concept to high‐intensity interval training (Pettitt 2016). With the $S^{\prime }$ equation, work bouts utilizing a fractional depletion of D′ that take into account $S_{\max}$, FI, and especially $\tau_{d}$, would yield more accurate *t*_LIM_ estimates involving partial D′ expenditure. Specifically, as the timing of W′ expenditure in cycling, and presumably D′ in running, is coupled tightly with the emergence of the $\dot{V}O_{2}$ slow component (Poole et al. 2016), exercise prescriptions requiring partial expenditures of D′ that take into account $\tau_{d}$ may lead to better predictable rates of metabolic responses as measured using $\dot{V}O_{2}$.

The bi‐exponential model of the present study represents a methodological advancement for the CS concept. Individual parameters of the bi‐exponential model may provide insight into characteristics of D′ not yet fully recognized. Of particular note, and perhaps the most novel finding revealing a unique aspect of D′, was the subject variability of $\tau_{d}$ and its independence from $S_{\max}$ and $A_{d}$. The variability of $\tau_{d}$ indicates that D′ represents more than a finite capacity; but rather, there exist individual differences concerning the rate at which D′ can be expended. Such information related to D′ cannot be gleaned from the traditional AOT or linear model techniques for deriving CS and D′ (i.e., D‐*t*_LIM_ and S‐1/*t*_LIM_ models).

The shuttle mode of the AOT offers a method of prescribing high‐intensity exercise that is more sport‐specific, with running involving frequent starts, stops, and turns. The length of the shuttle distance mandates changes in the parameters of high‐intensity running (e.g., $S_{\max}$, D′, and potentially CS) due to the frequency of 180° turns and needs to repetitively decelerate. Practitioners prescribing high‐intensity running based on the shuttle AOT should therefore keep in mind that the CS and D′ parameters are specific to the shuttle distance.

## Conflicts of Interest

There are no conflicts of interest. The results of the study are presented clearly, honestly, and without fabrication, falsification, or inappropriate data manipulation.
